# Evaluation of the effectiveness and adverse events of oxycodone as a pain-relieving agent in 103 dogs with tumors: a retrospective study

**DOI:** 10.1186/s12917-025-04987-3

**Published:** 2025-10-02

**Authors:** Byung-Gee Ko, Tae-Hyun Kim, Hyun Namkung, Hye-Gyu Lee, Young-Jun Kang, Ga-Hee Youn, Kyoung-Won Seo, Hwa-Young Youn, Min-Ok Ryu

**Affiliations:** Laboratory of Internal Medicine, Department of Veterinary Clinical Science, College of Veterinary Science, Seoul National University, Seoul, 08826 Republic of Korea

**Keywords:** Analgesic, Cancer, Canine, Combination, Metastasis, Pain

## Abstract

**Background:**

Oxycodone, a semi-synthetic opioid, has been widely used in human medicine for the management of tumor-related pain due to its potent analgesic properties. This study aimed to evaluate the efficacy and safety of oxycodone in managing tumor-related pain in dogs, addressing the current lack of research on effective analgesic options for canine cancer dogs. Medical records of 103 tumor-bearing dogs treated with oxycodone were retrospectively reviewed.

**Results:**

Oxycodone demonstrated an analgesic effect ranging from 23.1 to 63.6%, depending on the anatomical origin of the tumor. Improvement was observed in 67.7% of dogs with metastatic lesions. In the monotherapy group, the mean pain relief score increased from 8.73 ± 1.27 before treatment to 9.65 ± 1.53 after administration (*p* < 0.0001). In the combination therapy group, score rose from 8.75 ± 1.20 to 9.83 ± 1.87 (*p* = 0.0002). Across the entire canine patient cohort, the mean pain relief score increased from 8.74 ± 1.23 to 9.74 ± 1.70 following treatment (*p* < 0.0001). Adverse effects included lethargy (*n* = 6), diarrhea (*n* = 2), vomiting (*n* = 1), tachypnea (*n* = 1), and stargazing (*n* = 1). All were self-limiting and resolved after discontinuation of the drug.

**Conclusions and clinical importance:**

Oxycodone demonstrated an overall favorable analgesic profile and could be a well-tolerated and promising pain management solution, both as monotherapy and in combination therapy, for tumor-related pain in dogs, including those with advanced-stage tumors.

## Background

As the prevalence of chronic diseases increases, the proportion of older dogs in hospital attendance correspondingly rises [1, 2]. Chronic pain, particularly that associated with cancer, significantly reduces quality of life (QOL) in dogs [3]. Often, the clinical presentation of chronic pain in dogs is limited to subtle or mild changes in behavior, such as alterations in posture, temperament, vocalization, or movement, which can be challenging to detect due to their subtlety [4]. Pain assessment tools such as the Glasgow Composite Measure Pain Scale and the Canine Brief Pain Inventory are commonly used to evaluate and quantify pain in dogs [5]. Enhanced awareness of chronic pain in dogs, along with the development of assessment tools, has facilitated earlier recognition [6, 7]. Nonetheless, managing tumor-related pain in veterinary patients remains difficult due to the need for effective analgesia, minimal impact on systemic condition, and adjustable dosing.

The search for novel applications of analgesics in veterinary clinical practice remains ongoing. Effective pain relief is crucial for reducing veterinary patient mortality and morbidity and enhancing the overall QOL [3, 8]. In veterinary medicine, the long-term use of nonsteroidal anti-inflammatory drugs (NSAIDs) is limited by species-specific differences that raise concerns regarding the risk of acute renal failure and gastrointestinal side effects [9, 10]. Consequently, the veterinary field has witnessed an escalating reliance on opioid analgesics to address tumor-related pain concerns despite the occurrence of respiratory depression, nausea and vomiting, histamine release, constipation, and central nervous system excitation associated with the use of mu opioid agonists. However, aside from transdermal fentanyl patches, the use of opioids in outpatient settings remains limited due to challenges such as the difficulty of precise dose adjustments, especially in small dogs [11].

Efforts have been made to overcome the limitations associated with opioid delivery, which has led to the development of oral opioid formulations [12, 13]. Unlike in humans, where mu-opioid agonists such as methadone exhibit high oral bioavailability, methadone in dogs undergoes extensive hepatic first-pass metabolism when administered orally. However, co-administration with the CYP3A inhibitor ketoconazole has been reported to increase its bioavailability up to approximately 29% in one dog [14]. Additionally, oxycodone has demonstrated its efficacy in managing pain arising from diverse chronic conditions, including tumor-related pain and various chronic pain syndromes in human medicine [15, 16]. Oxycodone is a semi-synthetic opioid about 1.5–2 times more potent than morphine, with high oral bioavailability and efficacy for moderate to severe pain in humans [17]. As a result, a pharmacokinetic study on oral intake has been conducted to explore the potential use of oxycodone as an analgesic in dogs [12]. However, it has been shown that the plasma concentration of noroxycodone, an inactive metabolite, is higher than that of the parent drug, oxycodone, in dogs [18]. Recent pharmacokinetic studies have also reported oxycodone’s elimination half-life was 2.6 h, which may require frequent dosing. In an effort to overcome these limitations, studies on hydrocodone have also been conducted [19, 20]. Nevertheless, there remains considerable interest and expectation surrounding oxycodone’s potential for effective pain control in veterinary medicine.

To the best of our knowledge, there is a lack of research evaluating the efficacy and safety of oxycodone in veterinary medicine. The principal objective of this retrospective study was to evaluate the effectiveness of oxycodone in the management of tumor-related pain conditions while meticulously documenting the occurrence of associated side effects. This study aimed to assess the analgesic potential of oxycodone and its feasibility for use in managing tumor-related pain in veterinary medicine.

## Materials and methods

### Study design

A retrospective review of medical records was conducted with institutional approval, and client consent was obtained for data use.

### Animals

The medical records of dogs that received oxycodone between March 2015 and August 2023 at the Seoul National University Veterinary Medical Teaching Hospital were retrospectively reviewed. Inclusion criteria required caregiver-reported pain in dogs with measurable tumors confirmed by fine-needle aspiration, biopsy, or imaging (radiography, ultrasonography, CT, or MRI), followed by oxycodone prescription. Dogs with well-defined masses on imaging considered high risk for aspiration were also eligible. Dogs with comorbidities that could independently contribute to pain, such as osteoarthritis, pancreatitis, infections, recent surgical history (within three weeks), or trauma, were excluded based on physical examination, palpation, and, when indicated, relevant diagnostic tests including biomarkers. None of the dogs received appetite stimulants or physiotherapy during the oxycodone evaluation period. Additionally, cases lacking follow-up or where caregivers did not administer the prescribed medication were omitted.

### Data collection and clinical evaluation

Data collected from the medical records included breed, sex, age, diagnosis, tumor-affected organs causing pain, concurrent analgesics, oxycodone dosage and administration frequency, duration of oxycodone treatment, efficacy of oxycodone, and any adverse events associated with its use.

Pain sources were determined by the veterinarian based on owner descriptions in the medical charts and physical examinations. In cases where the pain was not clearly localized, the most active lesion or condition strongly suspected to be the cause of the pain was identified.

### Drug formulation and dosage

Oxycodone hydrochloride (Ocodon^®^, 5 mg tablet, Hana Pharm Co., Ltd., Seoul, Republic of Korea) is an immediate-release formulation of oxycodone and was prescribed uniformly to all dogs in this study. Oxycodone was administered perorally at a standardized dose of 0.3 mg/kg per administration, with a default dosing frequency of every 8–12 h [21–23]. However, the attending veterinarian adjusted the frequency as needed based on the dog’s condition and the owner’s circumstances, ranging from every other day to every 6 h. Tablets were prescribed according to the dog’s weight, and in some cases, tablets were crushed to adjust the dose.

### Assessment and scoring of pain relief and adverse effects

Tumor-related pain is a representative form of chronic pain, with severity ranging widely from subtle to severe. In veterinary medicine, various tools have been continuously proposed to assess tumor-associated pain [24, 25]. One of the most widely used scales for evaluating tumor-associated pain to date is the Canine Brief Pain Inventory (CBPI) [26]which has been primarily applied in cases of bone tumors. However, in our institution, the symptoms reported by owners through interviews or questionnaires were more consistent with those assessed by the Canine Symptom Assessment Scale [27]. These included pain, reduced activity, decreased appetite, changes in panting or respiratory patterns, vocalization, and difficulty sleeping. The majority of tumor-bearing dogs included in this study were considered geriatric, typically over 8 years of age. During CBPI assessments, which include items such as rising, walking, running, and climbing, most owners reported that they were unable to evaluate these aspects of mobility. Furthermore, the majority of owners perceived that inducing activities such as running or stair climbing solely for pain evaluation was unnecessary. Therefore, in addition to the items of the Glasgow Composite Measure Pain Scale [28]including appetite, activity, vocalization, and response to touch, we added sleep quality and respiratory rate, which were the most frequently reported pain-related responses by caregivers.

Following oxycodone administration, pain was assessed before and after treatment using owner-reported scores based on specific parameters, including physical activity levels, appetite, pain relief in response to manipulation, vocalization (such as crying, grunting, or screaming), respiratory rate, and both sleep duration and quality according to the modified tumor-related pain relief scoring system (Table 1) [6, 29, 30]. Pain assessment was statistically analyzed based primarily on evaluations conducted between days 7 and 14, considering the steady-state of oxycodone. In dogs who received long-term prescriptions, there was Little difference between the pain relief scores at day 14 and those obtained during subsequent follow-up. However, for dogs who discontinued oxycodone within 7 days, pain relief scoring was performed at the time of discontinuation. The sum score was calculated by adding the individual scores for activity, appetite, pain, vocalization, respiration rate, and sleeping disorder. Improvement was defined as an increase of at least 1 point in either an individual item or the sum score following oxycodone administration. The assessment of adverse drug reactions was conducted using the Naranjo algorithm [31]. Only adverse events with a total Naranjo algorithm score of 4 or higher were included.

**Table 1:** The modified tumor-related pain relief scoring system used in this study

	score	clinical correlation
activity	0	Depressed or non-responsive
	1	Indifferent or quiet
	2	Happy and bouncy
appetite	0	Force feeding and no interest in food or water
	1	Skipping or wanting only treats or people food
	2	Eating and drinking normally
pain	0	Growl or guard for manipulation
	1	Flinch or look round for manipulation
	2	Do nothing
vocalization	0	Groaning
	1	Crying or whimpering
	2	Quiet
resting respiratory rate	0	Panting or increased respiratory effort; RR ≥ 30/min
	1	Short and shallow breathing; RR ≥ 30/min
	2	normal respiration; RR < 30/min
sleep quality	0	Trouble falling asleep
	1	Frequent arousal
	2	Deep or sound sleep

*RR* Respiratory rate

The anatomic origin of pain was determined based on the location of the primary tumor in cases without metastasis. In cases of metastasis, the most likely pain-generating site at the time of oxycodone prescription was identified through canine patient history, physical examination, manipulation, and imaging, and a single site was selected for classification. Anatomic sites were classified as viscera, skin, mucosa, bone, muscle, nerve, and lymph nodes. Tumors originating in visceral organs with concurrent enlargement of visceral lymph nodes were categorized as visceral rather than nodal in origin.

### Treatment

This study was conducted retrospectively, and the prescription of medications was determined at the discretion of the attending veterinarian. Based on the pain scoring provided by the caregiver and the veterinarian’s clinical judgment of the dog, decisions were made regarding the discontinuation or reduction of previously prescribed analgesics, combination therapy, or the sole use of oxycodone. Simultaneously, the initial dosage and frequency of oxycodone administration were determined.

### Statistical analysis

Statistical analyses were performed using GraphPad Prism 10 software (GraphPad, CA, USA). To assess changes in pain relief scores within individual subjects, the Wilcoxon matched-pairs signed rank test was used to compare pre-treatment and post-treatment values for each subject. Statistical significance was set at *P* < 0.05.

## Results

### Veterinary patient information

A total of 113 dogs were deemed eligible, but 10 were excluded owing to comorbidities that could cause pain or a recent history of trauma or surgery, leaving 103 dogs in the study. The population comprised 44 neutered males, 43 neutered females, 12 intact females, and 4 intact males. The median age was 12 (range, 3–19 years), and the main breeds were Maltese (*n* = 28), Shih-tzu (*n* = 11), mixed breeds (*n* = 10), Yorkshire terriers (*n* = 8), Schnauzer (*n* = 7), Cocker spaniel (*n* = 7), and Poodles (*n* = 5). Information on the remaining breeds is provided in Table 2.

**Table 2:** Demographic and clinical characteristics of the tumor bearing dog cohort prescribed oxycodone for pain management

Breed	Akita (1), Beagle (1), Chihuahua (2), Cocker Spaniel (7), Dachshund (1), Jindo (3), Labrador retriever (3), Maltese (28), Miniature Pinscher (2), Mixed (10), Pekingese (1), Pomeranian (3), Pompitz (1), Miniature Poodle (5), Schnauzer (7), Scottish terrier (1), Shetland sheepdog (1), Shiba inu (1), Shih-tzu (11), Siberian Husky (1), Silky terrier (1), Spitz (3), Welsh Corgi (1), Yorkshire terrier (8)
The number of dogs (n) is indicated for each category, and breed distribution is shown in parentheses	

When classified according to cellular origin, the tumors were categorized as follows: carcinoma (*n* = 52, 50.5%), round cell tumor (*n* = 22, 21.3%), sarcoma (*n* = 19, 18.4%), neuroendocrine tumor (*n* = 2, 1.9%), and other (*n* = 8, 7.8%). The anatomical distribution of the tumors included viscera (*n* = 55, 54.4%), skin (*n* = 12, 11.7%), mucosa (*n* = 10, 9.7%), bone (*n* = 9, 8.7%), muscle (*n* = 7, 6.8%), nerve (*n* = 6, 5.8%), and lymph nodes (*n* = 4, 3.9%). A detailed list of specific diagnoses is provided in Table 3.

**Table 3:** Primary diagnosis at the time of oxycodone prescription: categorized by anatomic origin

Anatomic origin	*n*	Diseases in detail
Viscera	55	STS (3), HSA (4), Adrenal mass (1), Lymphoma (11), AGASAC (3), CCC (2), HCC (7), RA mass (2), Insulinoma (1), Extramedullary plasmacytoma (1), Intestinal adenocarcinoma (1), PuAC (1), Carcinoma metastasis (2), Pancreatic adenocarcinoma (1), PrAC (6), Renal (2) TCC (7)
Skin	12	Cutaneous lymphoma (3), Inflammatory MGT (5), Melanoma (1), STS (3)
Mucosa	10	Oral STS (1), Oral SCC (5), Oral melanoma (2), Nasal adenocarcinoma (1), Oral fibrosarcoma (1)
Bone	9	Carcinoma metastasis (4), OSA (3), STS (2)
Muscle	7	MGT (3), MCT (2), STS (1), Salivary gland adenocarcinoma (1)
Nerve	6	Intracranial tumor (2), Carotid body tumor (1), PNST (1), Carcinoma metastasis (1), Glioma (1)
Lymph node	4	Lymphoma (2), Carcinoma metastasis (2)

*STS* Soft tissue sarcoma, *HSA* Hemangiosarcoma, *AGASAC* Apocrine gland anal sac adenocarcinoma, *CCC* Cholangiocellular carcinoma, *HCC* Hepatocellular carcinoma, *RA* Right atrium, *PuAC* Pulmonary adenocarcinoma, *PrAC* Prostate adenocarcinoma, *TCC* Transitional cell carcinoma, *MGT* Mammary gland tumor, *STS* Soft tissue sarcoma, *SCC* Squamous cell carcinoma, *OSA* Osteosarcoma, *MCT* Mast cell tumor, *PNST* Peripheral nerve sheath tumor, *n* number of dogs

### Efficacy and adverse events of oxycodone

The duration of oxycodone administration ranged from a minimum of 2 days to a maximum of 472 days, with a median duration of 14 days. The initial dosing regimen consisted of once daily (*n* = 3, 2.9%), twice daily (*n* = 53, 51.5%), three times daily (*n* = 45, 43.7%), four times daily (*n* = 2, 1.9%), every other day (*n* = 1, 0.9%), and as needed (*n* = 1, 0.9%). Among the eight dogs who required dosage escalation, improvement was observed in two cases. No additional adverse events were reported following dosage adjustments.

Of the 103 dogs, 56 (54.4%) demonstrated improvement in the sum score following oxycodone administration. Specifically, following oxycodone administration, increased activity was observed in 30 (29.1%), enhanced appetite in 25 (24.3%), decreased pain in 35 (34%), reduced respiratory rates in 15 (14.6%), alleviation of sleep disturbances in 13 (12.6%) and reduced vocalization was noted in 6 (5.8%) dogs.

Adverse effects were observed in 10 dogs, including grade 1 lethargy (*n* = 6, 5.8%), grade 1 diarrhea (*n* = 2, 1.9%), grade 1 vomiting (*n* = 1, 0.9%), grade 1 tachypnea (*n* = 1, 0.9%) and stargazing (*n* = 1, 0.9%) followed by VCOG-CTCAE v2 [32]. Adverse events were reported between 3 and 35 days after the initiation of oxycodone, with a median time to onset of 10 days.

The dog who received oxycodone for the longest duration was treated for 472 days. During the treatment period, renal and hepatic parameters were regularly monitored and remained within the reference range. Except for an elevation in renal values associated with urolithiasis shortly before death, no evidence of oxycodone-induced renal or hepatic dysfunction was observed.

### Relationship between the efficacy of oxycodone and anatomic origin

The improvement rate of oxycodone based on anatomic origin, as determined by the sum score, was as follows—viscera (*n* = 35, 63.6%), skin (*n* = 2, 23.1%), mucosa (*n* = 4, 40%), bone (*n* = 6, 66.7%), muscle (*n* = 4, 57.1%), nerve (*n* = 3, 50%), and lymph node (*n* = 2, 50%), with an overall improvement rate of (*n* = 56, 54.4%). The distribution of observed pain-related symptoms across the six pain assessment categories was as follows—viscera 164 cases, skin 34 cases, mucosa 26 cases, bone 30 cases, muscle 22 cases, nerve 19 cases, and lymph node 14 cases. On average, each dog reported pain in three categories.

According to the sum score criteria, anatomic origins exhibiting an improvement rate of 50% or higher included viscera, bone, muscle, nerve, and lymph node, whereas improvement rate below 50% were observed in the skin and mucosa. When anatomic origin was not considered, oxycodone demonstrated improvements in activity (*n* = 30, 43.5%), appetite (*n* = 25, 39.7%), pain (*n* = 35, 42.1%), vocalization (*n* = 6, 31.6%), respiration rate (*n* = 15, 38.5%), and sleeping disorder (*n* = 13, 43.3%). Detailed values for individual assessment parameters are presented in Table 4.

**Table 4:** Data on the improvement rates to oxycodone treatment across different tumor anatomical origins, based on the pain relief scoring system used in this study, including its use within multimodal analgesic protocols

		viscera	skin	mucosa	bone	muscle	nerve	lymph node	total
n		55	12	10	9	7	6	4	103
activity	pre-treatment abnormal (n)	40	8	6	7	4	5	3	69
	post-treatment improved (n)	23	1	2	3	0	1	0	30
	improvement rate	57.50%	12.50%	33.30%	42.80%	0%	20%	0%	43.50%
appetite	pre-treatment abnormal (n)	37	8	7	3	4	2	2	63
	post-treatment improved (n)	19	2	1	1	0	1	1	25
	improvement rate	51.30%	25%	14.30%	33.30%	0%	50%	50%	39.70%
pain	pre-treatment abnormal (n)	43	8	6	9	7	6	4	83
	post-treatment improved (n)	19	2	3	5	4	2	0	35
	improvement rate	44.20%	25%	50%	55.60%	57.10%	33.30%	0%	42.10%
vocalization	pre-treatment abnormal (n)	5	4	2	3	2	2	1	19
	post-treatment improved (n)	1	2	1	2	0	0	0	6
	improvement rate	20%	50%	50%	66.70%	0%	0%	0%	31.60%
respiration rate	pre-treatment abnormal (n)	25	3	1	5	2	3	2	39
	post-treatment improved (n)	10	1	1	3	0	0	0	15
	improvement rate	40%	33.30%	100%	60%	0%	0%	0%	38.50%
sleep quality	pre-treatment abnormal (n)	14	3	4	3	3	1	2	30
	post-treatment improved (n)	6	1	1	1	2	0	2	13
	improvement rate	42.80%	33.30%	25%	33.30%	66.70%	0%	100%	43.30%
sum score	pre-treatment abnormal (n)	55	12	10	9	7	6	4	103
	post-treatment improved (n)	35	2	4	6	4	3	2	56
	improvement rate	63.60%	23.10%	40%	66.70%	57.10%	50%	50%	54.40%

Pre-treatment abnormality was defined as a score of 0 or 1 for each individual parameter and a total sum score of less than 12. Post-treatment improved was defined as an increase of at least 1 point from the pre-treatment score

*n* number of dogs

### Comparison of oxycodone efficacy between combination therapy and monotherapy

Among the 103 dogs prescribed oxycodone, 51 (49.5%) received it as monotherapy, while 52 (50.5%) were administered oxycodone in combination with other analgesics. The most frequently co-administered drugs were gabapentin 18 (34.6%), tramadol 15 (28.8%), piroxicam 15 (28.8%), fentanyl 8 (15.4%), firocoxib 7 (13.5%), celecoxib 3 (5.8%), meloxicam 1 (1.9%), hydromorphone 1 (1.9%), and buprenorphine 1 (1.9%). A single additional analgesic was prescribed in 38 (36.9%), two additional analgesics in 11 (10.7%), and three additional analgesics in 3 (2.9%). The specific drug combinations are detailed in Table 5.

**Table 5:** Oxycodone monotherapy and combination therapy regimen categories

Concurrent medication		
Number of concurrent medication	Treatment	n (%)
None (*n* = 51, 49.5%)		51 (49.5%)
1 medication (*n* = 38, 36.9%)	Opioids (Tramadol PO)	5 (4.9%)
	Opioids (Fentanyl patch)	4 (3.9%)
	NSAIDs (Firocoxib PO)	3 (2.9%)
	NSAIDs (Piroxicam PO)	10 (9.7%)
	NSAIDs (Celecoxib PO)	2 (1.9%)
	NSAIDs (Meloxicam PO)	1 (0.97%)
	Gabapentinoids (Gabapentin PO)	13 (12.6%)
2 medications (*n* = 11, 10.7%)	Opioids (Tramadol PO, Buprenorphine patch)	1 (0.97%)
	Opioids (Tramadol PO) + NSAIDs (Piroxicam PO or Firocoxib PO or Celecoxib PO)	5 (4.9%)
	Opioids (Tramadol PO) + Gabapentinoids (Gabapentin PO)	2 (1.9%)
	Opioids (Fentanyl patch) + NSAIDs (Firocoxib PO)	1 (0.97%)
	Opioids (Fentanyl patch) + NSAIDs (Piroxicam PO)	1 (0.97%)
	NSAIDs (Piroxicam PO) + Gabapentinoids (Gabapentin PO)	1 (0.97%)
3 medications (*n* = 3, 2.9%)	Opioids (Tramadol PO, fentanyl patch) + Gabapentinoids (Gabapentin PO)	1 (0.97%)
	Opioids (Tramadol PO, Hydromorphone IV) + NSAIDs (Firocoxib PO)	1 (0.97%)
	Opioids (Tramadol PO) + NSAIDs (Firocoxib PO) + Gabapentinoids (Gabapentin)	1 (0.97%)

*IV* Intravenous, *NSAIDs* Non-steroidal anti-inflammatory drugs, *PO* Per oral, *n* number of dogs

**Fig. 1:**
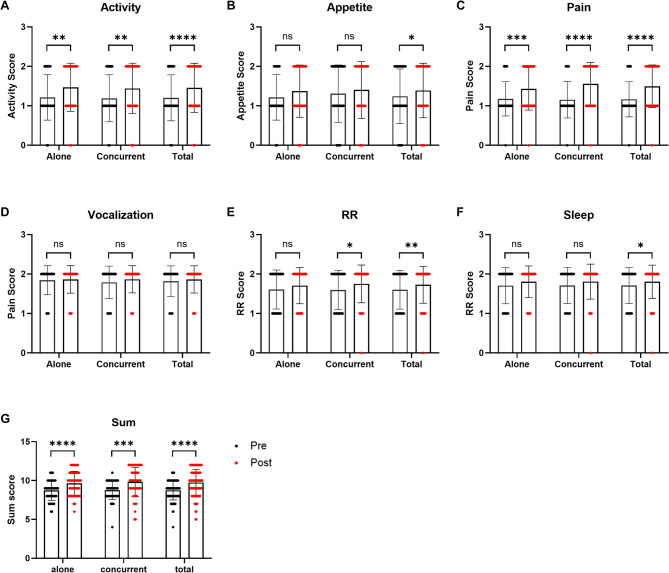
Comparison of the efficacy of oxycodone monotherapy and combination therapy using the pain relief scoring system of this study. Changes in individual pain-related scores before and after oxycodone treatment in dogs receiving oxycodone alone (Alone), oxycodone with concurrent medications (Concurrent), and the total population (Total). The evaluated parameters include (**a**) activity, (**b**) appetite, (**c**) pain, (**d**) vocalization, (**e**) respiration rate (RR), (**f**) sleep, and (**g**) sum score. Each bar represents the mean ± standard deviation of the scores before (black) and after (red) treatment. *P*-values were calculated using the Wilcoxon matched-pairs signed rank test to assess within-subject differences between pre-treatment and post-treatment values. Statistical significance was defined as *p* < 0.05. *, **, ***, **** indicates *p* < 0.05, *p* < 0.01, *p* < 0.001, and *p* < 0.0001 compared to the pre-treatment value, respectively

The total sum score, reflecting the overall clinical response, demonstrated significant improvement across all groups, including monotherapy (*p* < 0.0001), combination therapy (*p* = 0.0002), and the total study population (*p* < 0.0001). The average difference in pain relief scores between pre- and post-treatment was 0.92 for monotherapy, 1.07 for combination therapy, and 1.0 for the total group. These findings are visually represented in Fig. 1.

### Relationship between the efficacy of oxycodone and metastasis

**Fig. 2:**
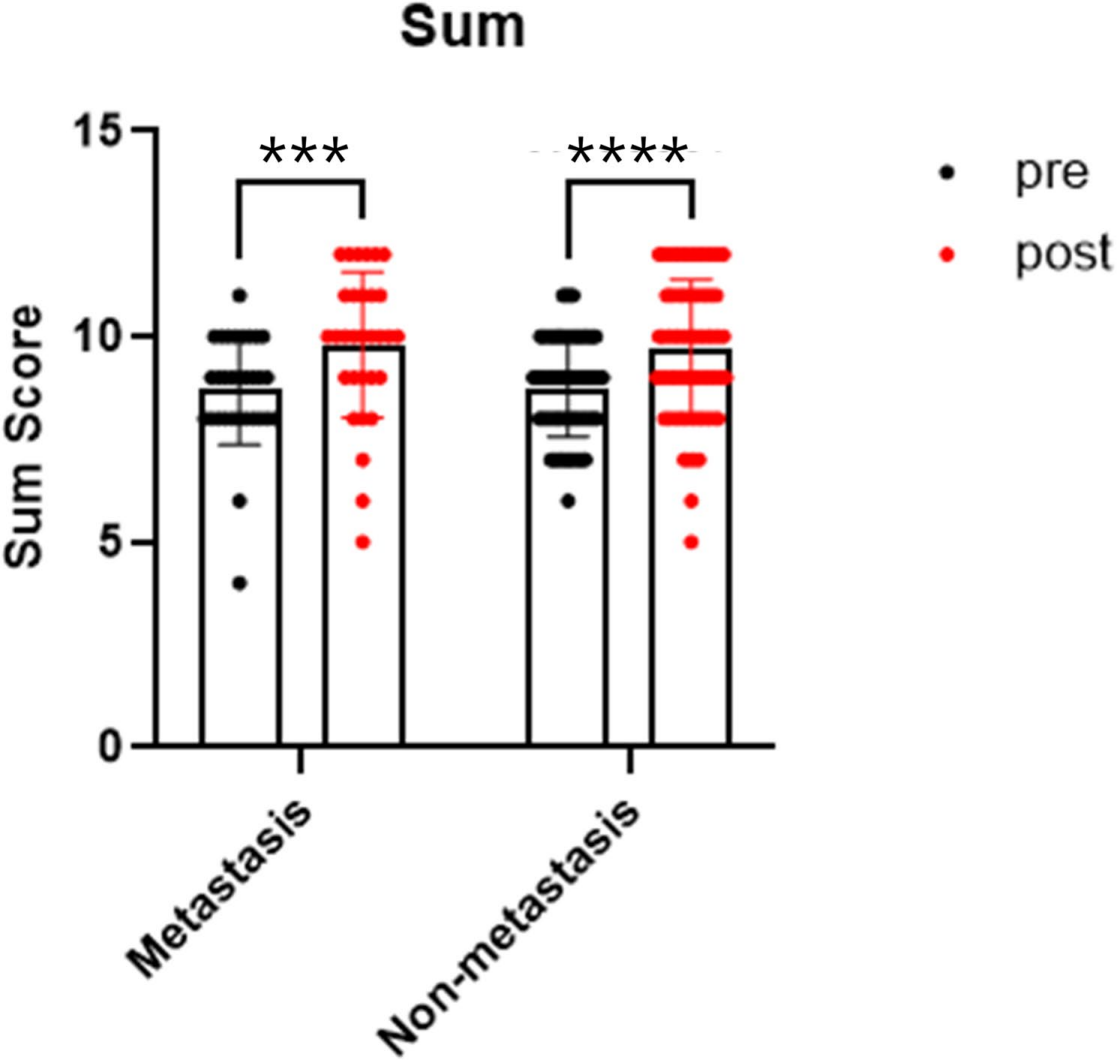
Comparison of oxycodone efficacy in metastatic and non-metastatic dogs using the pain relief scoring system of this study. Changes in total sum scores before and after oxycodone treatment in dogs with metastatic lesions and those without. Each bar represents the mean ± standard deviation of the scores before (black) and after (red) treatment. The length of each dot and line increased in proportion to the number of dogs associated with the corresponding score. To evaluate within-subject changes in total sum scores, the Wilcoxon matched-pairs signed rank test was used to compare pre-treatment and post-treatment values for each subject. Statistical significance was defined as *p* < 0.05. Graphs are presented as mean ± standard deviation. ^***, ****^ indicates *p* < 0.001 and *p* < 0.0001 compared to the pre-treatment value, respectively

A total of 31 dogs had metastasis, while 72 dogs were without metastasis. Based on the distribution of metastatic sites, involvement was identified in the lymph nodes (*n* = 9, 29%), lungs (*n* = 11, 35.5%), liver (*n* = 9, 29%), spleen (*n* = 8, 25.8%), bones (*n* = 5, 16.1%), and peritoneum (*n* = 3, 9.7%). In the metastatic cohort, the mean pre-treatment sum score was 8.7, which improved to 9.8 post-treatment (*p* < 0.001). In the non-metastatic cohort, the pre-treatment sum score was 8.7, increasing to 9.7 after treatment (*p* < 0.0001). Sum score improvement was observed in 21 metastatic dogs and 34 non-metastatic dogs. These findings are visually represented in Fig. 2.

## Discussion

This retrospective study provides new insights into the efficacy and safety of oxycodone, a novel opioid formulation, in pain management for canine tumor patients. In human medicine, oxycodone is considered a viable alternative to morphine, demonstrating high oral bioavailability and substantial efficacy across various pain types, particularly neuropathic and somatic pain [33]. It has shown effectiveness in reducing pain intensity, improving functional disability, enhancing performance metrics, and overall enhancing QOL [34–37]. Furthermore, it led to a substantial reduction in Numeric Rating Scale values in dogs experiencing chronic visceral pain due to tumors [38].

Tumors are an inevitable cause of chronic pain in elderly dogs. Previous studies have shown that the prevalence of tumors increases exponentially from the age of seven, regardless of breed or body weight. In this study, a diverse range of breeds was enrolled, from small breeds such as Maltese and Yorkshire Terriers to medium and large breeds such as Cocker Spaniels, Labrador Retrievers, and Jindos. The age range of tumor bearing dogs was between 3 and 19 years, with a median age of 12 years. Among them, 96 dogs (*n* = 96, 93.2%) were older than seven years. This trend likely reflects the aging demographics and prevalence of comorbidities (e.g., chronic kidney disease, chronic enteropathy) in veterinary patients, which limit long-term NSAID use [9], while the mild adverse effects of oxycodone observed in 10 (9.7%) out of 103 dogs allowed continued prescription.

Oxycodone was administered to tumors located in various anatomical sites, including the viscera, skin, mucosa, bone, muscle, nerves, and lymph nodes. Pain alleviation was observed in tumors of diverse cellular origins, such as carcinoma, sarcoma, round cell tumors, and neuroendocrine tumors. Notably, the most frequently treated and significantly improved anatomical site was the viscera (*n* = 55, 63.6%), suggesting that oxycodone may provide targeted analgesic effects for tumor-associated visceral pain in veterinary patients. This aligns with previous human studies that have highlighted the distinct pharmacological properties of oxycodone in managing visceral pain, especially compared to other opioids. Oxycodone is a full mu opioid receptor agonist that also has notable affinity for kappa opioid receptors, a property that contributes to its enhanced efficacy in managing visceral pain. This is attributed to the role of kappa receptors in the transmission pathway of visceral pain and the increased expression of these receptors in the brain and spinal cord as pain becomes chronic [39–41]. Oxycodone has demonstrated analgesic effects on skin lesions, such as those seen in scleroderma, in humans. However, in this study cohort, a notably low improvement rate of 23.1% was observed in dogs with cutaneous tumors [42]. This may be due to differences in the mechanisms of pain caused by cutaneous tumors and autoimmune diseases, suggesting that oxycodone may not provide sufficient analgesic effects in cases of cutaneous tumor involvement. Oxycodone showed only a 14.3% improvement in the appetite domain for dogs with mucosal involvement, which may be attributed to the fact that 9 out of the 10 dogs had lesions on the oral mucosa. This made it difficult to distinguish between appetite loss due to insufficient analgesic effect and that caused by the anatomical location of the lesion.

Oxycodone was administered in nearly equal proportions as monotherapy (*n* = 51, 49.5%) and in combination therapy (*n* = 52, 50.5%). Previous studies have consistently investigated the efficacy and safety of combining opioids with NSAIDs or gabapentinoids [43–46]. Combination therapy has been expected to provide additional analgesic effects through mechanisms distinct from opioids while also allowing for dose-sparing effects, potentially reducing adverse effects. In this study, NSAID (*n* = 25, 24.2%) and gabapentin (*n* = 18, 17.4%) were the most commonly co-administered medications; however, no significant differences in analgesic efficacy were observed between monotherapy and combination therapy. These findings suggest that, within the limitations of this retrospective study, oxycodone may provide comparable analgesic efficacy and tolerability whether used alone or as part of a multimodal analgesic regimen.

Moreover, our findings revealed that oxycodone produced an improvement rate exceeding 60% even in dogs with advanced-stage tumors metastasized to the lungs, bones, or lymph nodes. It displayed broad analgesic efficacy and extended to metastatic lesions beyond the primary organs, consistent with findings reported in human studies [47]. In the cases of human metastatic prostate cancer, the degree of pain has been directly linked to overall survival, making the analgesic effect of oxycodone in metastatic cancer particularly promising [48].

Human studies use standardized tools such as the Visual Analogue Scale for Pain, Numeric Rating Scale for Pain, McGill Pain Questionnaire, Chronic Pain Grade Scale, and Measure of Intermittent and Constant Osteoarthritis Pain to evaluate chronic pain severity and treatment efficacy [49]. These instruments provide a quantitative framework for evaluating the efficacy of pain management interventions. In veterinary medicine, the Glasgow Composite Pain Scale, primarily intended for acute pain assessment (typically post-operative), and the Canine Brief Pain Inventory, designed for osteoarthritis, have limitations in evaluating tumor-related pain in dogs [6, 50, 51].

Within the context of our study, we employed pain (reaction to manipulation), activity, appetite, and vocalization based on the Glasgow Composite Pain Scale. Additionally, we included respiratory rate and sleep pattern, which were the most frequently reported concerns by caregivers, for evaluation. Notably, the most substantial improvement was noted in activity, constituting 43.5%, followed by improvements in sleep disturbances at 43.3%, pain at 42.1%, and modest improvements in appetite, respiration, and vocalization, at 39.7%, 38.5%, and 31.6%, respectively. It should be emphasized that in older adults, symptoms such as vocalization or sleep disturbances may be erroneously attributed to cognitive dysfunction syndromes [52]. However, the efficacy of oxycodone treatment serves to underscore these manifestations as integral components of a pain profile.

Moreover, beyond the outlined advantages, oxycodone effectively mitigated the practical challenges posed by conventional intravenous or transdermal patch opioid formulations and addressed insufficient research concerning the safety and efficacy of extended-term prescriptions [53, 54]. Our study implemented oxycodone for up to 472 days. Although the number of long-term cases was limited, no serum chemistry abnormalities related to hepatic or renal function were observed during prolonged oxycodone administration. However, as most patients received the medication for a relatively short duration, the long-term safety of oxycodone could not be adequately assessed in this study.

Adverse effects associated with oxycodone administration in humans have been frequently documented and include constipation, nausea, drowsiness, dizziness, vomiting, and pruritus, which are commonly reported in descending order of occurrence [16, 55]. Incidences of addiction were rare, and patients exceeding a daily dosage of 60 mg were systematically tapered off to mitigate potential withdrawal symptoms [56]. Although this study noted mild gastrointestinal clinical signs, lethargy, and increased sleep duration, instances of constipation and pruritus were notably absent. However, given the veterinary patient cohort and treatment duration, it is imperative to maintain ongoing surveillance for the potential emergence of these issues in future cases, necessitating continued monitoring during prescription. The median prescribed dose in this investigation was 0.3 mg/kg, which closely aligns with the recommended initial dosage of 10 mg in humans based on body surface area. Dosage frequency ranged from every 4 to 6 h, depending on pain severity, but most of the veterinary patients were initially prescribed twice daily dosing. Given the potential for dosage escalation and increased frequency, further research is warranted to explore the adverse effects and clinical signs of withdrawal documented in existing literature.

This study has several limitations. First, as a retrospective study based on medical records, we could not objectively control the prescribed frequency of oxycodone. Moreover, the lack of standardized protocols for dose adjustments and formulation introduced inherent variability. Second, the small sample sizes for each anatomic site subgroup may result in limited statistical significance when comparing anatomic-specific effects. The effects of age, breed, body weight, and sex on the efficacy of oxycodone were not separately analyzed. Third, since analgesics were administered in a clinical setting, we were unable to include a placebo group. Fourth, the enrolled dogs’ heterogenous population was based on clinician judgment rather than predefined inclusion and exclusion criteria, which may have introduced bias. Finally, the degree of pain relief in this study was assessed solely through subjective parameters such as owner-reported outcomes, and we were unable to incorporate objective measures. Future large-scale prospective studies are needed to provide a clearer evaluation of the analgesic effects of oxycodone.

## Conclusions

To the best of our knowledge, this is the first retrospective study to demonstrate the efficacy and adverse effects of oxycodone in dogs with tumors. This study helps establish the rationale for prescribing oral oxycodone for managing tumor-related pain in dogs, based on its validated efficacy and tolerability. Oxycodone significantly improved activity, pain severity, and sleep patterns with modest improvements in appetite, respiration and vocalization. It has shown an efficacy rate exceeding 60%, even in dogs with advanced-stage tumors. The adverse effects were mild and manageable, emphasizing the potential of oxycodone as a long-term pain management agent in veterinary medicine. We anticipated that oxycodone would serve as a potentially valuable analgesic for tumor-related pain, filling a longstanding gap in veterinary pain management. Further research is recommended to explore long-term safety and efficacy to ensure optimal pain relief in canine oncology patients.

## Data Availability

The authors declare that all the data supporting the findings of this study are available within this article, its supplementary information files, or are available from the corresponding author, who has all relevant data, upon reasonable request.

## Abbreviations

CBPI: Canine brief pain inventory
NSAIDs: Non-steroidal anti-inflammatory drugs
QOL: Quality of life
